# Serous Pleuritic Effusion Treated by Incision

**Published:** 1898-02-26

**Authors:** 


### SEROUS PLEURITIC EFFUSION TREATED BY INCISION.

At the last meeting of the Clinical Society Dr. West related the case of a man, aged thirty-nine, whose right chest had been full of fluid for twelve months. The patient had a strong family history of phthisis and probably was phthisical himself. During a few weeks after admission the chest was tapped three times, large quantities of fluid being removed, which, however, quickly reaccumulated. It was then (fifteen months after the beginning of his illness) decided to lay open his chest. A large quantity of fluid was evacuated at the time of the operation. No rib was excised. The patient improved greatly, but the temperature rose and the discharge became purulent. The lung, however, quickly expanded, and at the end of a week was in close contact with the ribs everywhere. It was not possible to leave the tube out, as the track extended a long way, nearly to the spine. The discharge very much diminished—to about two ounces a day—but it contained tubercle bacilli. Reference was made to other cases of serous effusion, in which, when the chest had refilled after repeated tappings, an incision had been made; good results, in fact complete cure, having followed. Dr. West thought that excision of the ribs should not be practised as a routine, but only when found necessary for complete drainage. On the surgical side this was criticised, and the removal of rib was advocated; while on the medical side it was suggested that tubercle was less apt to spread in lung that was collapsed. Nevertheless, the proof of the pudding is in the eating, and we cannot but feel that Dr. West is wise in removing fluid, even by incision, when it persistently reappears after tapping. The more one looks to fresh air and an outdoor life as curative influences in the treatment of tuberculosis, the more one feels impelled to remove any condition which stands in the way of a patient availing himself of such means of cure.

